# Tumor Suppressors TSC1 and TSC2 Differentially Modulate Actin Cytoskeleton and Motility of Mouse Embryonic Fibroblasts

**DOI:** 10.1371/journal.pone.0111476

**Published:** 2014-10-31

**Authors:** Elena A. Goncharova, Melane L. James, Tatiana V. Kudryashova, Dmitry A. Goncharov, Vera P. Krymskaya

**Affiliations:** Airways Biology Initiative, Pulmonary, Allergy & Critical Care Division, Department of Medicine, Perelman School of Medicine, University of Pennsylvania, Philadelphia, PA, United States of America; University of Birmingham, United Kingdom

## Abstract

TSC1 and TSC2 mutations cause neoplasms in rare disease pulmonary LAM and neuronal pathfinding in hamartoma syndrome TSC. The specific roles of TSC1 and TSC2 in actin remodeling and the modulation of cell motility, however, are not well understood. Previously, we demonstrated that TSC1 and TSC2 regulate the activity of small GTPases RhoA and Rac1, stress fiber formation and cell adhesion in a reciprocal manner. Here, we show that *Tsc1^−/−^* MEFs have decreased migration compared to littermate-derived *Tsc1^+/+^* MEFs. Migration of *Tsc1^−/−^* MEFs with re-expressed TSC1 was comparable to *Tsc1^+/+^* MEF migration. In contrast, *Tsc2^−/−^* MEFs showed an increased migration compared to *Tsc2^+/+^* MEFs that were abrogated by TSC2 re-expression. Depletion of TSC1 and TSC2 using specific siRNAs in wild type MEFs and NIH 3T3 fibroblasts also showed that TSC1 loss attenuates cell migration while TSC2 loss promotes cell migration. Morphological and immunochemical analysis demonstrated that *Tsc1^−/−^* MEFs have a thin protracted shape with a few stress fibers; in contrast, *Tsc2^−/−^* MEFs showed a rounded morphology and abundant stress fibers. Expression of TSC1 in either *Tsc1^−/−^* or *Tsc2^−/−^* MEFs promoted stress fiber formation, while TSC2 re-expression induced stress fiber disassembly and the formation of cortical actin. To assess the mechanism(s) by which TSC2 loss promotes actin re-arrangement and cell migration, we explored the role of known downstream effectors of TSC2, mTORC1 and mTORC2. Increased migration of *Tsc2^−/−^* MEFs is inhibited by siRNA mTOR and siRNA Rictor, but not siRNA Raptor. siRNA mTOR or siRNA Rictor promoted stress fiber disassembly in TSC2-null cells, while siRNA Raptor had little effect. Overexpression of kinase-dead mTOR induced actin stress fiber disassembly and suppressed TSC2-deficient cell migration. Our data demonstrate that TSC1 and TSC2 differentially regulate actin stress fiber formation and cell migration, and that only TSC2 loss promotes mTOR- and mTORC2-dependent pro-migratory cell phenotype.

## Introduction

Mutations of tumor suppressor genes *tuberous sclerosis complex 1 (TSC1) and TSC2* are linked to the pathobiology of hamartoma syndrome Tuberous Sclerosis (TSC) and pulmonary lymphangioleiomyomatosis (LAM) [1]–[5]. TSC is a genetic disease characterized by hamartomas in multiple organs including the kidneys, brain, skin and heart and is associated with abnormal neuronal pathfinding in the developing brain [2], [5]. Pulmonary LAM, a rare disease that can be sporadic or associated with TSC, is characterized by the neoplastic growth of smooth-muscle like lesions in the lungs, destruction of the lung parenchyma, loss of pulmonary function, and is associated with increased occurrence of renal angiomyolipomas [6]. In addition to abnormal proliferation, smooth muscle-like cells from LAM lungs have increased motility and invasiveness [7], and LAM nodule recurrence was reported after single-lung transplantation in patients without renal angiomyolipoma [8], suggesting a metastatic nature of cells with mutational inactivation of TSC1/TSC2. The specific roles of TSC1 and TSC2 in cell migration and invasiveness, however, are not clear, and underlying mechanisms are not well understood.

The major breakthrough in understanding the functions of TSC1 and TSC2 came with identifying that TSC2 binds TSC1 via its N-terminal domain and forms the TSC1/TSC2 tumor suppressor complex that acts as a negative regulator of the mammalian target of rapamycin (mTOR) complex 1 (mTORC1), a key regulator of cell growth, proliferation, metabolism, and autophagy [9]–[11]. Tumor suppressor function of TSC1/TSC2 is exerted by TSC2 that acts as GTPase Activating Protein (GAP) for small GTPase Rheb via its C-terminal domain [12]. TSC2 GAP inhibits Rheb, leading to Rheb-dependent inhibition of mTORC1, its downstream effectors S6 kinase 1 (S6K1)-ribosomal protein S6, and suppression of cell growth and proliferation [12].

Studies from our group and others demonstrated that TSC1 activates RhoA GTPase and interacts with ERM proteins, and identified TSC2 as upstream regulator of TSC1-RhoA signaling pathway [13], [14]. We also reported that TSC1 and TSC2 regulate the activity of RhoA and Rac1 GTPases in a reciprocal manner: TSC2 loss induces TSC1-dependent inhibition of Rac1 associated with RhoA activation [14]. Thus, evidence suggests that TSC1 and TSC2 regulate Rho family GTPases and are involved in actin cytoskeleton and focal adhesion remodeling, but their roles in cell migration and invasiveness are not well understood.

Interestingly, the TSC1-binding N-terminal domain of TSC2 is sufficient for the regulation of actin stress fibers as well as RhoA and Rac1 activity [14], but is not involved in the regulation of mTORC1-S6 signaling and DNA synthesis, suggesting that TSC1/TSC2 effects on actin cytoskeleton or Rho GTPases are independent of its anti-proliferative function [14]. In addition to rapamycin-sensitive mTORC1 (mTOR-Raptor), mTOR forms a catalytic core of the rapamycin-insensitive mTORC2 (mTOR/Rictor) regulating Akt [15], PKCα [16], Rac1 [17] and RhoA [18]–[20]. The role of TOR in the regulation of the actin cytoskeleton has been well known in yeast [21]. Studies have now established that mTOR regulates the actin cytoskeleton through Rac1 [16], [17] as a part of the rapamycin-insensitive mTORC2.

The goal of this study was to identify whether TSC1 or TSC2 has an effect on cell migration and morphology and whether these effects are mediated by mTORC1 or mTORC2. By using genetically modified MEFs, NIH 3T3 fibroblasts, and siRNA- and expression-based approaches, we found that TSC1 and TSC2 have differential effects on the actin cytoskeleton, cell morphology, and migration. Importantly, while TSC1 loss impairs cell migration, loss of TSC2 promotes stress fiber assembly and increases cell migration in mTOR- and mTORC2-dependent way.

## Materials and Methods

### Cell culture

NIH 3T3 fibroblasts were purchased from ATCC (ATCC CCL-92) and maintained in DMEM supplemented with 10% FBS, penicillin/streptomycin, and L-Glutamine. *Tsc1^−/−^* and *Tsc2^−/−^* MEFs with littermate matched wild-type controls *Tsc1^+/+^* and *Tsc2^+/+^* MEFs were generously provided by Dr. David Kwiatkowski from Harvard Medical School [22]; *Tsc1^−/−^* MEFs are spontaneously immortalized; *Tsc2^−/−^* MEFs are immortalized by p53 deletion [22]. *Rictor^−/−^* MEFs were generously provided by Dr. David Sabatini, from Massachusetts Institute of Technology. MEFs were maintained in DMEM supplemented with 10% FBS and were serum deprived in 0.1% FBS for 24 h prior to each experiment. Eker rat TSC2-null ELT3 cells were a generous gift from Dr. Cheryl L. Walker, of the Institute for Biosciences and Technology at Texas A&M Health Science Center, and were maintained as described in previously published studies [14], [23].

### Cell migration assay

Cell migration was examined using a Boyden chamber apparatus as we described previously [24]–[27]. Serum-deprived cells were briefly trypsinized by 0.05% trypsin/0.53 mM EDTA, centrifuged at 900 rpm for 10 min and resuspended in serum-free media supplemented with 0.5% FBS. Cells (5×10^4^) were then placed into the upper wells of the Boyden chamber fitted with an 8-µm pore membrane, coated with Vitrogen (100 µg/ml). Agonists or vehicle in serum-depleted media supplemented with 0.5% FBS were added to the lower chambers. Cells in the Boyden chamber were incubated for 4 h at 37°C in a 5% CO_2_ incubator. Non-migrated cells were scraped off; the membrane was fixed with methanol, stained with Hemacolor stain set (EM Industries, Inc., Gibbstown, NJ), and scanned. Cell migration was analyzed using the Gel-Pro analyzer program (Media Cybernetics, Silver Spring, MD).

### Wound assay and live imaging of wound closure

Cells, plated on chamberslides, were wounded by scraping a 10 µl-pipette tip through the cell monolayer; then gently washed with PBS, incubated with fresh media supplemented with 2% FBS for 2 h, followed by supravital analysis [14]. Supravital analysis was performed in the micro-incubator model CSMI (Harvard Apparatus, Holliston, MA) with constant 37°C temperature on a Nikon TE300 Inverted Microscope (Nikon Instruments Inc., Melville, NY) equipped with an Evolution QEi digital video camera (Media Cybernetics, Silver Springs, MD) under 100X magnification for 8.3 h. Images were taken every 10 minutes, and were analyzed using Image-Pro Plus 5.0.0.39 software (Media Cybernetics, Silver Springs, MD).

### Cell invasion assay

Cell invasiveness was examined using the Cultrex 96 Well basement membrane extract (BME) Cell Invasion Assay Kit (Trevigen Inc., Gaithersburg, MD). BME consists of basement membrane components that include collagen IV, laminin, and fibronectin extra-cellular matrix (ECM) proteins. Briefly, 50,000 serum-deprived MEFs were placed into separate wells of the Cultrex cell invasion chamber, pre-coated with BME gel; cell culture media was placed into the lower wells of chamber. After 24 h of incubation, non-invaded cells were washed off; cells that invaded the ECM were incubated with Cell Dissociation/Calcein AM Solution, and the fluorescence of this solution was measured using a fluorescent 96-well plate reader (485 nm excitation, 520 nm emission).

### Microinjection

Microinjection was performed using Eppendorf Microinjection System (Hamburg, Germany) as described previously [14], [23]. Eighteen hours after injection, cells were subjected to immunocytochemical assays.

### Immunocytochemistry

Cells were washed 3 times with PBS, fixed with 3.7% paraformaldehyde for 15 min, treated with 0.1% Triton X-100 for 30 min at room temperature, and blocked with 2% BSA in PBS [14], [23]. After incubation with rhodamine phalloidin (Molecular Probes, Eugene, OR), or primary and then secondary antibodies conjugated with either Alexa Fluor488, Alexa Fluor594, or Alexa Fluor633 cells were mounted in Vectashield mounting medium (Vector Laboratories, Burlingame, CA). Immunostaining was analyzed using the Leica TSC SP2 scanning laser confocal microscopic system, Nikon Eclipse TE2000-E microscope equipped with Evolution QEi digital video camera, or Nikon Eclipse E400 microscope equipped with Nikon Coolpix 995 digital camera under 600X magnification. 3-D analysis was performed using Z-series images taken with z interval 0.1 µm, which then were 3-D deconvoluted using AutoDebur + AutoVisualize Software 9.3 (Auto Quant Imaging Inc., Watervliet, NY).

### Immunoblot analysis

Cells were transfected with siRNA TSC1, siRNA TSC2, and control siGLO RISC-Free siRNA using RNAiFect transfection reagents (Qiagen, Valencia, CA) or with mammalian vectors expressing TSC1, TSC2, or via empty plasmid using Effectene transfection reagent (Qiagen, Valencia, CA). 48 h post-transfection, protein levels were detected by immunoblot analysis with specific anti-TSC1 and anti-TSC2 antibodies; then immunoblot analysis with specific antibodies to detect indicated proteins was performed as described previously [14], [18].

### Statistical analysis

Statistical analysis of F-actin staining or immunostaining was performed by using Nikon Eclipse E400 microscope images taken at 200X magnification followed by quantitative analysis using Gel-Pro Analyzer Software. Statistical analysis was performed using StatView 5 software. Data points from individual assays represent the mean values ± SE. Statistically significant differences among groups were assessed with the analysis of variance (ANOVA) (Bonferroni-Dunn test), with values of P<0.05 sufficient to reject the null hypothesis for all analyses. All experiments were designed with matched control conditions within each experiment to enable statistical comparison as paired samples.

## Results

### TSC1 and TSC2 differentially modulate actin cytoskeleton and cell morphology

**Figure 1:**
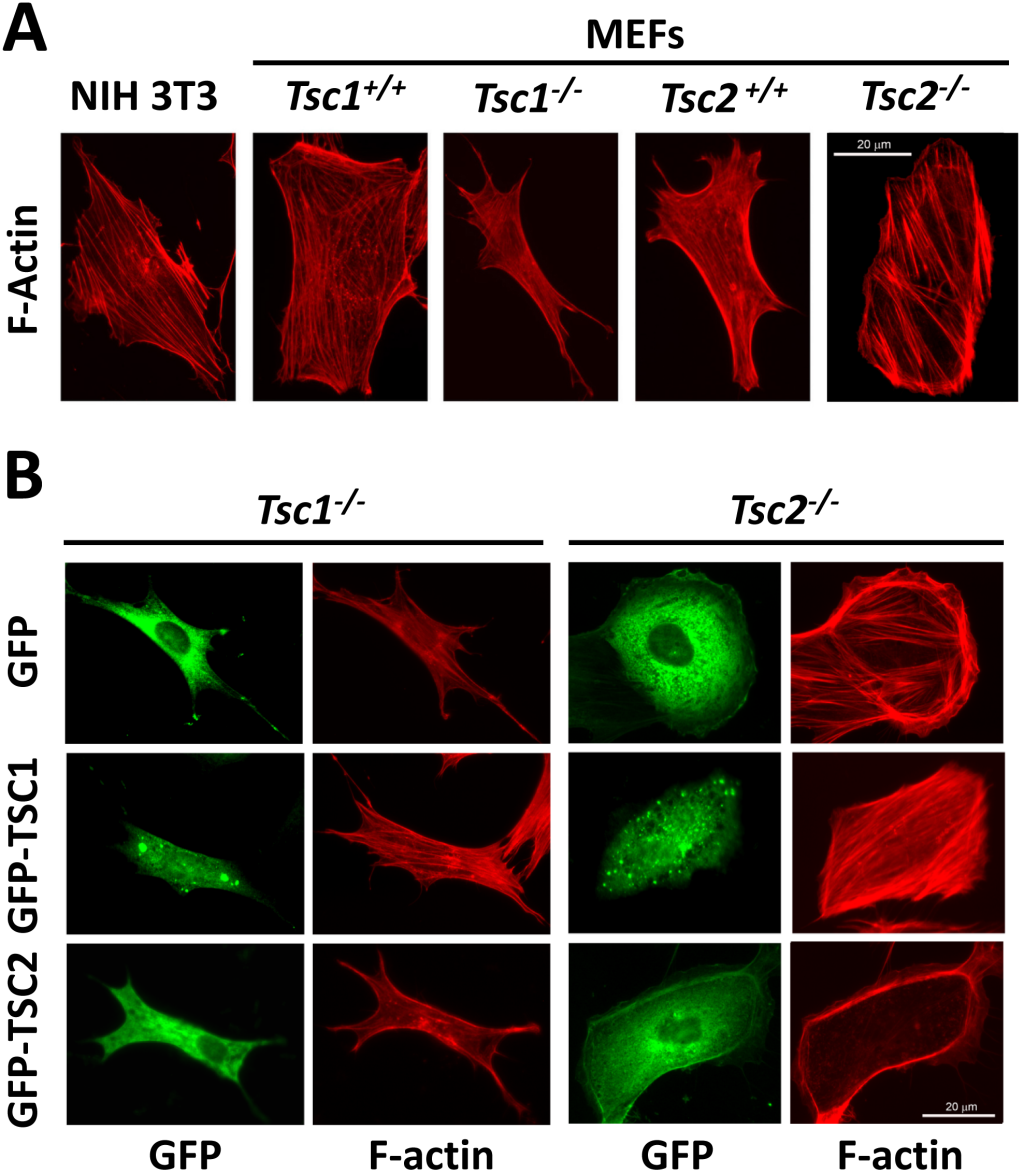
Effects of TSC1 and TSC2 on actin cytoskeleton. A: F-actin staining of serum-deprived NIH 3T3 fibroblasts and matched *Tsc1^+/+^*, *Tsc1^−/−^* and *Tsc2^+/+^*, and *Tsc2^−/−^* MEFs. B: F-actin staining (red) of *Tsc1^−/−^* and *Tsc2^−/−^* MEFs transfected with either GFP-TSC1, GFP-TSC2, or GFP. Representative images of two separate experiments were taken using a Nikon Eclipse TE-2000E microscope at 200x magnification. Scale bar, 20 µm.

Aberrant migration and invasiveness are characteristics of tumor cells with increased metastatic potential. Given the pivotal role of the actin cytoskeleton in the morphology, motility and invasiveness of cells, we performed rhodamine-phalloidin staining of serum-deprived NIH 3T3 fibroblasts, *Tsc1^−/−^* and *Tsc2^−/−^* MEFs, and littermate-derived *Tsc1^+/+^* and *Tsc2^+/+^* MEFs to determine the effects of TSC1 and TSC2 deficiency on the actin cytoskeleton. In contrast to stress fibers seen in NIH 3T3 fibroblasts and wild type MEFs, which are typical for mesenchymal cells, *Tsc1^−/−^* MEFs had thin-shaped bodies and thin extended filopodia protrusions with linear thin stress fibers (Fig. 1A). In contrast, *Tsc2^−/−^* MEFs have round or ellipsoid shapes and showed thick stress fibers predominantly attached to cortical actin at lamellipodia (Fig. 1A).

**Figure 2:**
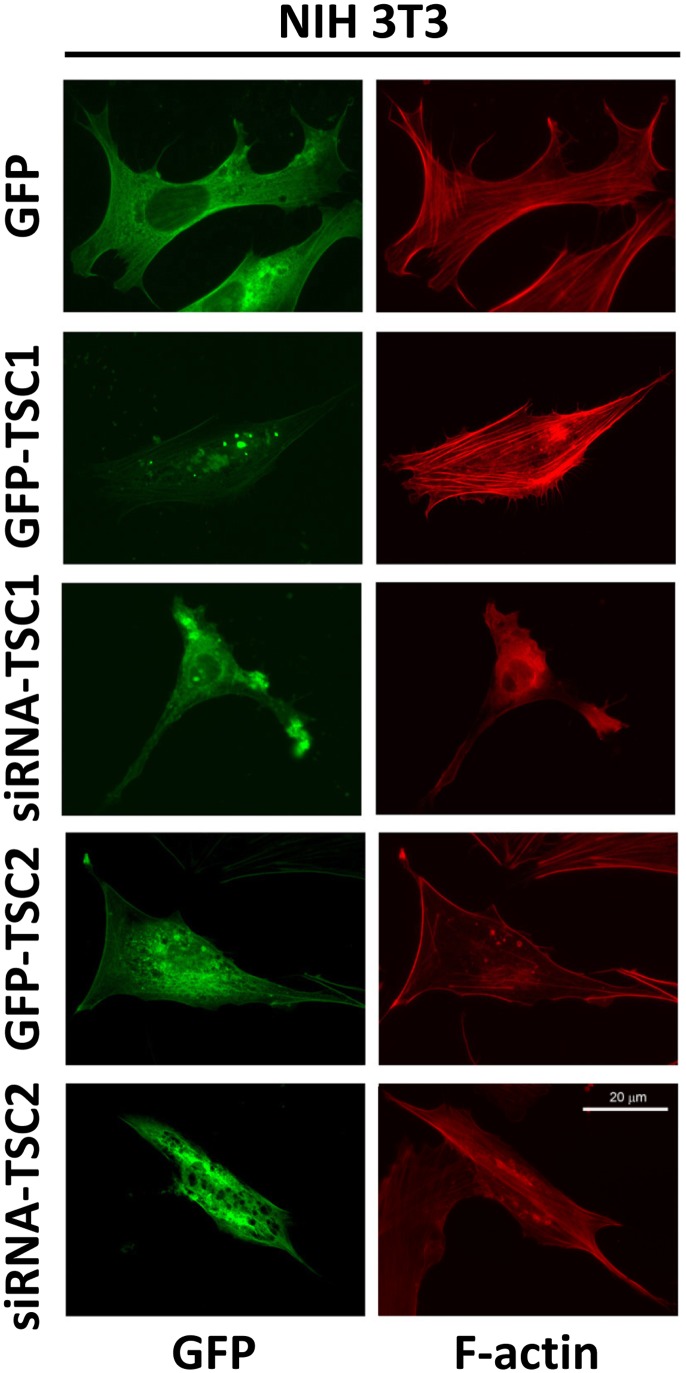
Effects of expression and siRNA-induced down-regulation of TSC1 and TSC2 in NIH 3T3 fibroblasts on actin cytoskeleton. Serum-deprived NIH 3T3 fibroblasts were transfected with GFP-TSC1, GFP-TSC2, or control GFP or microinjected with siRNA TSC1 or siRNA TSC2 and GFP followed by rhodamine phalloidin staining to detect F-actin (red) and immunostaining with anti-GFP antibody (green) to identify transfected or injected cells. Scale bar, 20 µm.

To further analyze the effects of TSC1 and TSC2 on the actin cytoskeleton, we performed rhodamine-phalloidin staining of *Tsc1^−/−^* and *Tsc2^−/−^* MEFs transfected with GFP-TSC1 and GFP-TSC2, respectively. Re-expression of TSC1 in *Tsc1^−/−^* MEFs promoted stress fiber formation (Fig. 1B, middle panel); similarly, expression of TSC1 in *Tsc2^−/−^* MEFs further promoted stress fiber formation and the attenuation of cortical actin (Fig. 1B, middle panel). Re-expression of GFP-TSC2 in *Tsc2^−/−^* MEFs promoted stress fiber disassembly and the enhanced formation of cortical actin (Fig. 1B, lower panel). In *Tsc1^−/−^* MEFs transfected with GFP-TSC2, actin was predominantly localized at the edges of cells forming cortical fibers (Fig. 1B, lower panel). As seen in Figure 2, in NIH 3T3 fibroblasts, GFP-TSC1 also promoted stress fiber formation, while siRNA TSC1 promoted stress fiber disassembly. In contrast, the transfection of NIH 3T3 fibroblasts with TSC2 promoted stress fiber disassembly and the formation of cortical actin, while cells with siRNA TSC2 have a thin shape and thin actin fibers (Fig. 2). Collectively, these data demonstrate that TSC1 and TSC2 differentially modulate stress fiber formation and the actin cytoskeleton.

**Figure 3:**
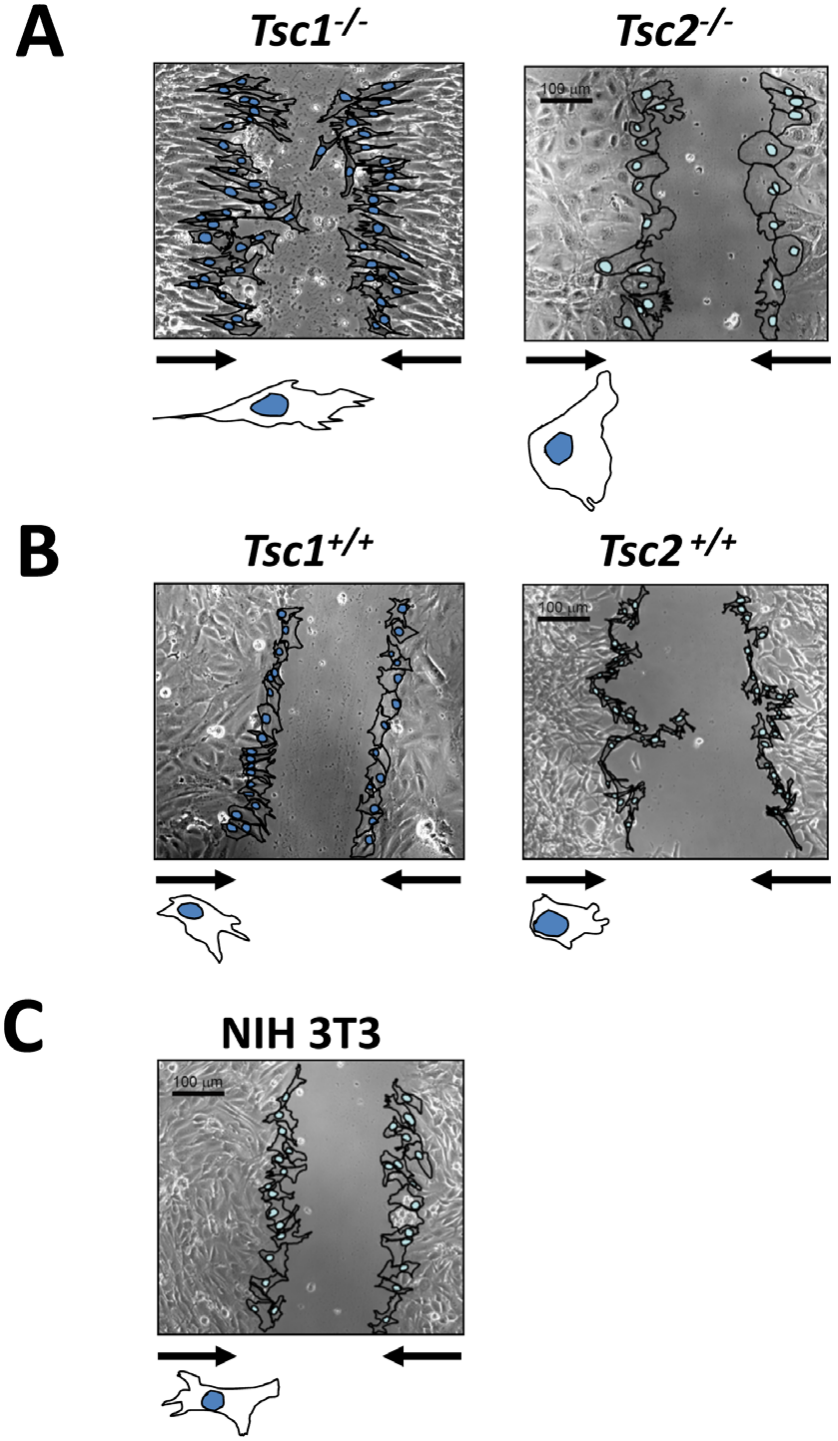
Morphology and dynamics of *Tsc1^−/−^* and *Tsc2^−/−^* MEFs during wound closure. Phase-contrast micrographs of cell motility during wound closure at 4 h after wound scraping. Arrows indicate direction of cell movement. Images were taken using a Nikon Eclipse TE2000-E microscope at 100X magnification in the phase contrast channel. Images are representative from three independent experiments. Scale bar, 100 µm.

The differences in the actin cytoskeleton are indicative of dynamic changes in cell morphology. Thus, we compared the morphology of *Tsc1^−/−^* and *Tsc2^−/−^* MEFs with littermate-matched wild type MEFs and NIH 3T3 fibroblasts during wound closure. Cell monolayers were serum-deprived and a wound assay was subsequently performed for 4 h in the presence of 2% FBS. As seen in Figure 3A, the morphology of migrating *Tsc1^−/−^* MEFs and *Tsc2^−/−^* MEFs were markedly different not only compared to those of wild type MEFs (Fig. 3B) and NIH 3T3 fibroblasts (Fig. 3C), but also between each other. In contrast to wild type MEFs and 3T3 fibroblasts that showed typical fibroblast morphology having irregular shape with a few protrusions towards wound closure, *Tsc1^−/−^* MEFs had a stretched shape with multiple filopodia-like extensions at the leading edge and thin extended cell shape of retraction in the rear (Fig. 3A, left panel). In contrast, motile *Tsc2^−/−^* MEFs showed broad lamellipodium at the front and round-like shape at the rear (Fig. 3A, right panel), as seen in the larger images of *Tsc2^−/−^* cells at higher magnification shown in Figure 1A and Figure 1B. Additionally, *Tsc2^−/−^* MEFs show phenotypic differences in apparent large cell size compared to *Tsc2^+/+^* MEFs (Figure 3A). Indeed, increased cell size due to activation of mTOR signaling was demonstrated in [28]. These data show that loss of TSC1 or TSC2 differentially modulate dynamic changes in cell morphology during motility.

### TSC1 and TSC2 differentially modulate migration and invasiveness

**Figure 4:**
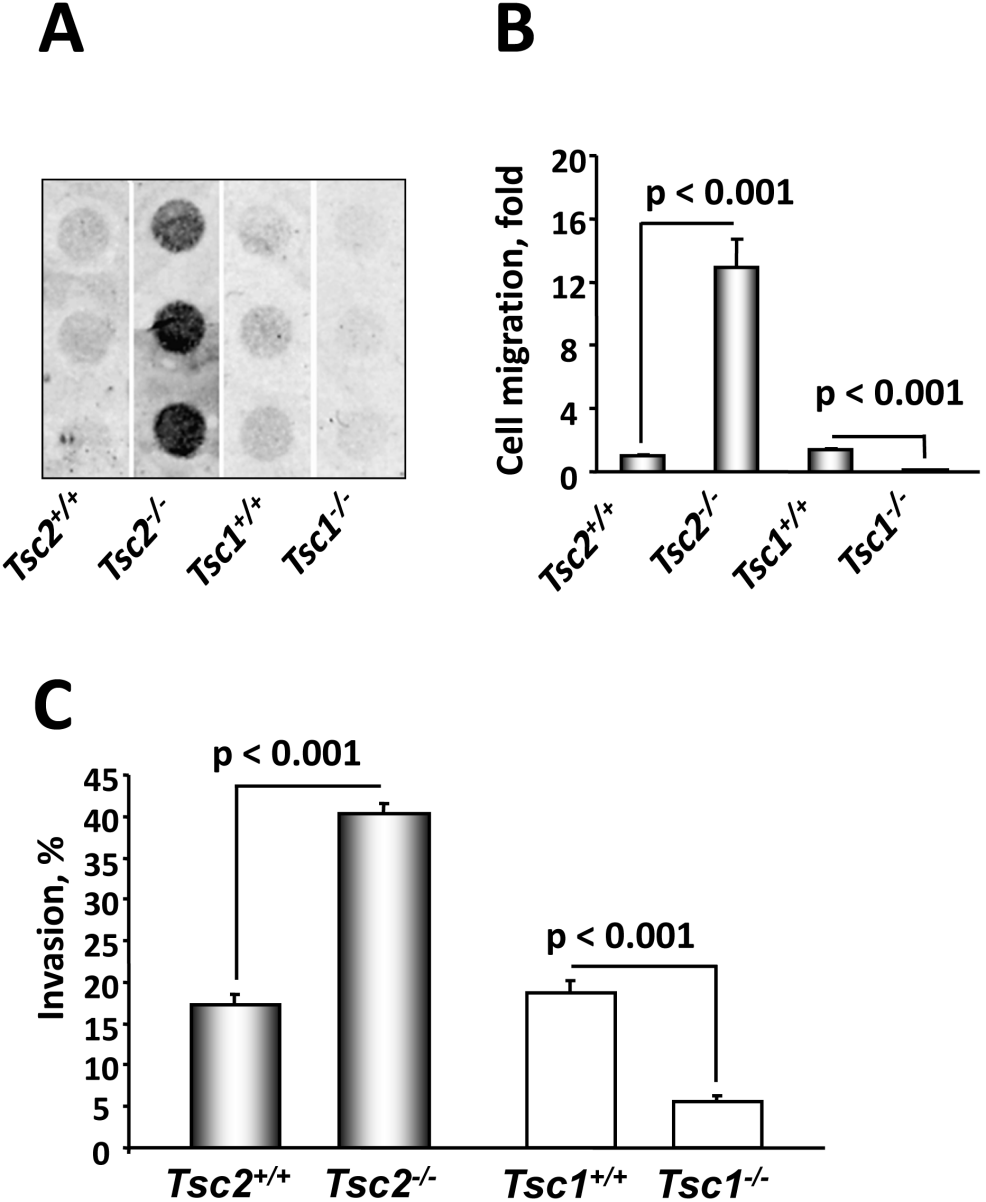
TSC1 and TSC2 differentially regulate migration and invasiveness. A: Representative membrane showing migration of serum-deprived *Tsc2^+/+^, Tsc2^−/−^, Tsc1^+/+^,* and *Tsc1^−/−^* MEFs. Serum-deprived cells were placed on collagen-saturated membranes in serum-free medium, and allowed to migrate in the Boyden chamber for 4 h in the absence of any stimuli. Then membranes were fixed, stained with Hemacolor stain set, and analyzed using Gel Pro software. B: Statistical analysis of migration experiments. Data represent mean values ± SE from measurements performed in triplicate from six separate experiments by ANOVA (Bonferroni-Dunn test). Basal migration of *Tsc2^+/+^* cells was taken as 1 fold. C: *Tsc2^−/−^* MEFs have increased invasiveness. Invasiveness of serum-deprived *Tsc2^+/+^, Tsc2^−/−^, Tsc1^+/+^,* and *Tsc1^−/−^* MEFs was analyzed using the Cultrex 96 Well BME Cell Invasion Assay kit according to manufacturer’s protocol. Data represent the percentage of invaded cells per total number of cells taken as 100%. Data represent mean values ± SE from two independent experiments by ANOVA (Bonferroni-Dunn test).

**Figure 5:**
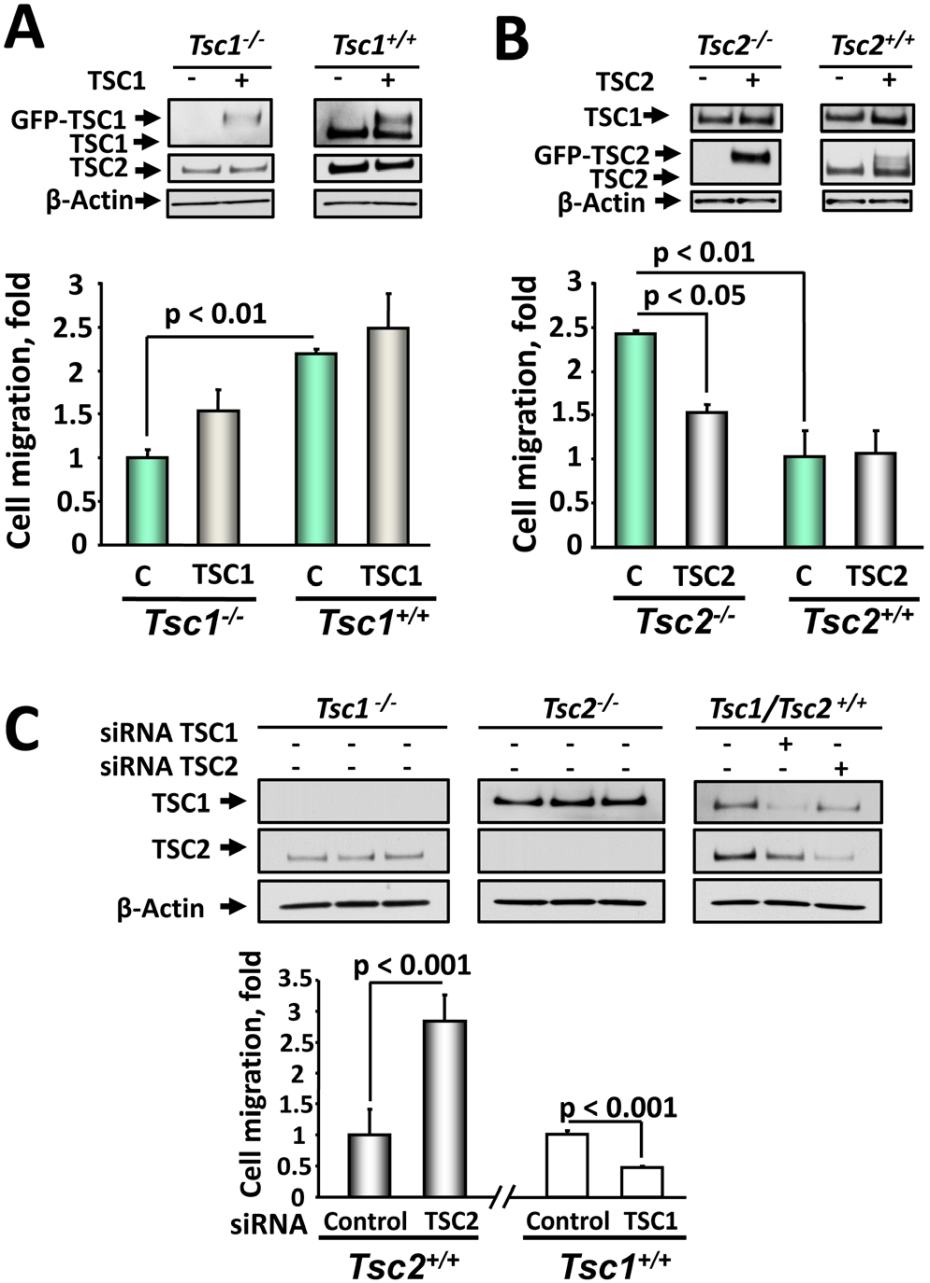
TSC1 and TSC2 re-expression or siRNA-induced knock-out validate their differential role in regulating cell migration. A: Re-expression of TSC1 rescues *Tsc1^−/−^* MEFs migration. MEFs were transiently transfected with TSC1 for 48 h, serum deprived followed by migration assay. B: Re-expression of TSC2 inhibits *Tsc2^−/−^* MEF migration. *Tsc2^+/+^* and *Tsc2^−/−^* MEFs were transiently transfected with TSC2, serum deprived followed by migration assays. TSC1 and TSC2 re-expression were confirmed by immunoblot analysis of equalized in protein content samples. Migration of *Tsc1^−/−^* (A) and *Tsc2^−/−^* MEFs (B) transfected with control plasmid was taken as 1 fold. Data represent mean values ± SE from two different experiments with three replicates for each condition by ANOVA (Bonferroni-Dunn test). C: Downregulation of TSC2, but not TSC1, promotes migration of wild type MEFs. *Tsc2^+/+^* and *Tsc1^+/+^* MEFs were transfected with siRNA TSC1, siRNA TSC2, and control siRNA. 48 h post-transfection, migration assays were performed. Protein levels were detected by immunoblot analysis with specific anti-TSC1 and anti-TSC2 antibodies under the same experimental conditions. Migration of wild type *Tsc1^+/+^* (right) or *Tsc2^+/+^* MEFs (left) transfected with siGLO RISC-Free siRNA was taken as 1 fold. Data represent mean values ± SE from measurements performed in triplicate from two separate experiments.

To assess the effects of the observed changes in actin cytoskeleton and cell morphology on their migratory behavior, we compared migratory and invasive potential of *Tsc1^−/−^* and *Tsc2^−/−^* MEFs and their littermate-derived wild type controls [18], [22], [27]. We found that both migration and invasiveness of serum-deprived unstimulated *Tsc2^−/−^* MEFs were markedly higher compared to *Tsc2^+/+^* MEFs (Fig. 4). Surprisingly, in contrast to *Tsc2^−/−^* cells, *Tsc1^−/−^* MEFs showed decreased migration and invasiveness compared to *Tsc1^+/+^* MEFs (Fig. 4). Increased migration rate of *Tsc2^−/−^* MEFs was inhibited by TSC2 re-expression (Fig. 5B). The decreased migration of *Tsc1^−/−^* MEFs was rescued by re-expression of TSC1 (Fig. 5A), which was only partial, potentially due to limitations of transient transfection and of using a whole population of transfected and non-transfected cells in migration assay. Immunoblot analysis of whole cell lysates equalized in protein content performed under the same experimental conditions demonstrated that in *Tsc1^−/−^* MEFs, TSC1 is absent, while there is a detectable TSC2 (Fig. 5A). Interestingly, expression of GFP-TSC1 also increased endogenous level of TSC2, showing that relative levels of TSC1 and TSC2 correlate with endogenous levels of TSC1 and TSC2 in *Tsc1^+/+^* MEFs (Fig. 5A). Similarly, expression of TSC2 in *Tsc2^−/−^* MEFs induced upregulation of TSC1 (Fig. 5B). These data support observation that loss of TSC1 or TSC2 leads to a decrease in protein levels of its binding partner and affects its localization [29].

**Figure 6:**
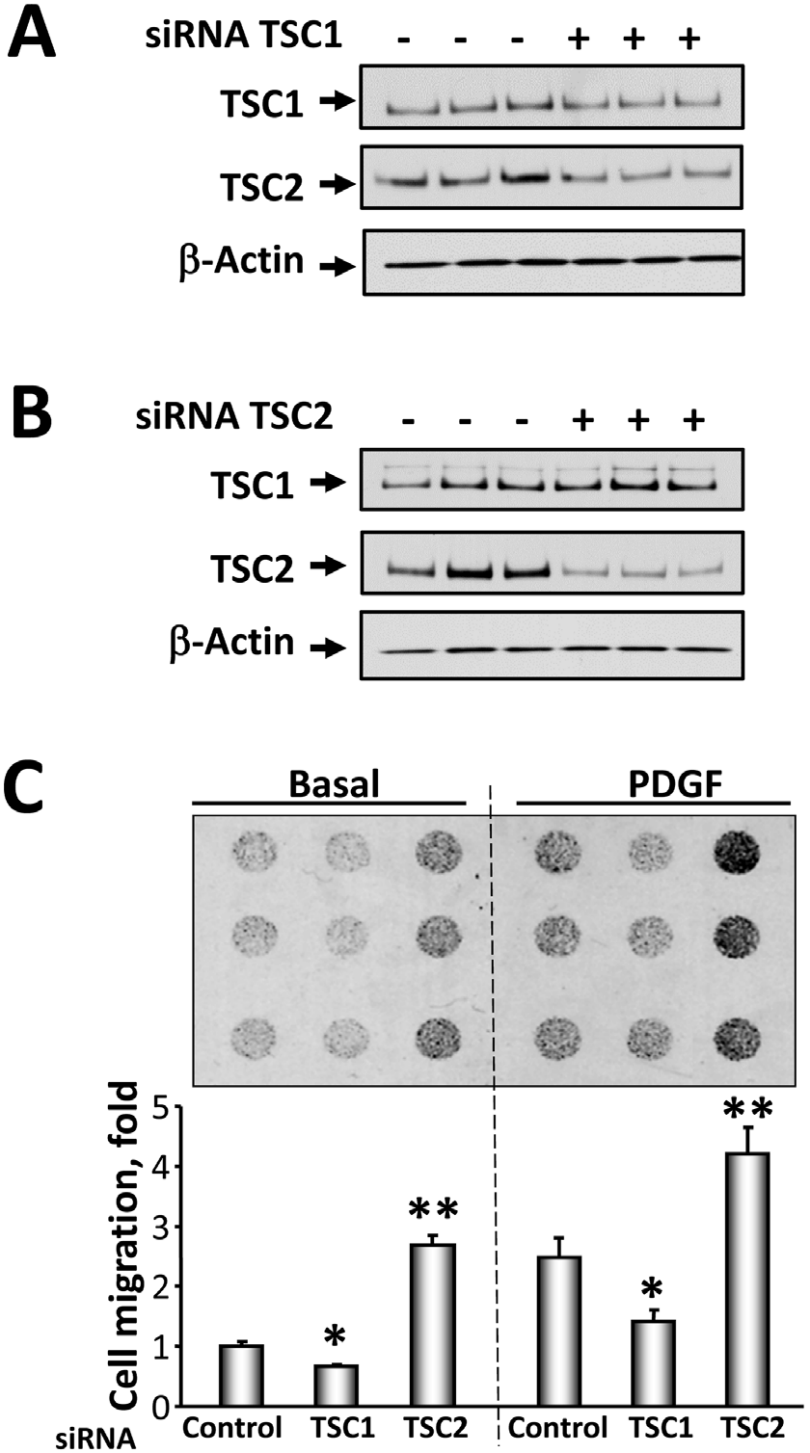
siRNATSC1 and siRNATSC2 induce opposite effects on NIH 3T3 fibroblast migration. Cells were transfected with siRNA TSC1 (A), siRNA TSC2 (B), or control siRNA. 48 h post-transfection, protein levels were detected by immunoblot analysis with anti-TSC1 or anti-TSC2 antibodies. C, Upper panel: Representative image of hemacolor-stained membrane with migrated NIH 3T3 fibroblasts for 4 h. 3T3 fibroblasts were transfected with siRNA TSC1, siRNA TSC2, and siGLO RISC-Free siRNA as control cells, serum-deprived followed by migration assay in the presence or absence of 10 ng/ml PDGF performed in triplicate for each experimental condition. C, Lower panel: Statistical analysis of NIH 3T3 cell migration. Data represent mean values ± SE from two independent experiments, six repetitions in each experiment. *P<0.01 for siRNA TSC1 vs. control siRNA, **P<0.001 for siRNA TSC2 vs. control siRNA by ANOVA (Bonferroni-Dunn).

To further validate a differential effect of TSC1 and TSC2 on cell migration, we investigated the effect of siRNA-induced down-regulation of TSC1 and TSC2 on the migration of wild type MEFs and NIH 3T3 fibroblasts. We found that TSC1 depletion in wild type *Tsc1^+/+^* MEFs attenuated cell migration (Fig. 5C). In contrast, siRNA TSC2 promoted *Tsc2^+/+^* MEF migration compared to cells transfected with control siRNA (Fig. 5C). Similarly, siRNA-induced TSC2 knock-down (Fig. 6A) increased basal and PDGF-induced migration of NIH 3T3 fibroblasts (Fig. 6B and 6C) while siRNA TSC1 (Fig. 6A) attenuated both basal and PDGF-induced NIH 3T3 migration compared to cells transfected with control siRNA (Fig. 6B and 6C). Collectively, these data demonstrate that TSC1 and TSC2 differentially modulate cell migration.

### mTOR and Rictor, but not Raptor, are required for stress fiber formation and migration of *Tsc2^−/−^* MEFs

**Figure 7:**
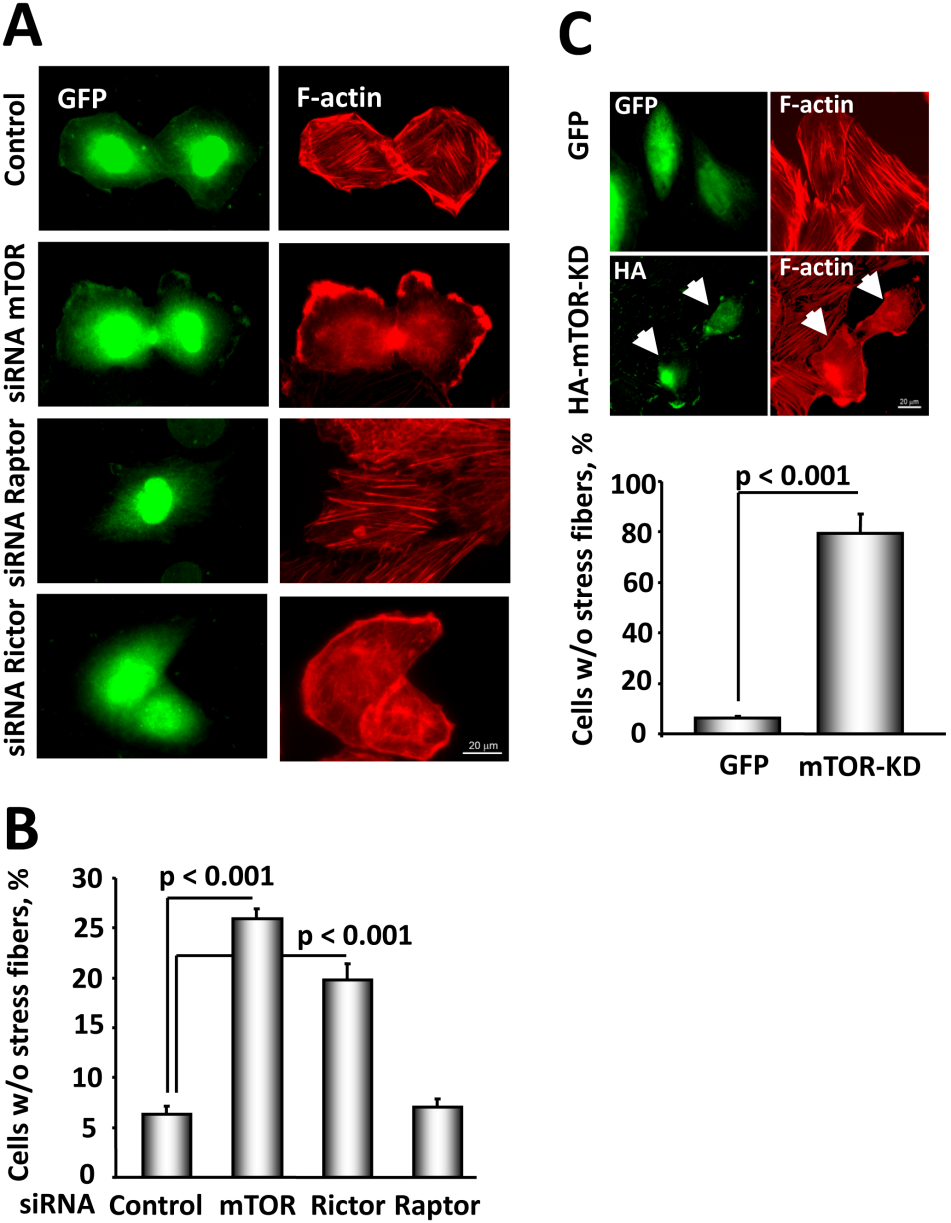
mTOR and Rictor, but not Raptor, modulate stress fiber formation in TSC2-null ELT3 cells. A: Cells were co-microinjected with siRNA mTOR, siRNA Rictor, siRNA Raptor, or control siRNA, and GFP to detect microinjected cells, serum-deprived followed by F-actin staining. Images were taken using a Nikon Eclipse TE-2000-E Microscope at 400X magnification. Scale bar, 20 µm. B: Statistical analysis. Data represent the percentage of cells without stress fibers per total number of microinjected cells taken as 100%. Data represent mean values ± SE by ANOVA (Bonferroni Dunn). C: mTOR activity is required for stress fiber formation in TSC2-null cells. Cells transfected with HA-tagged mTOR-KD were serum-deprived followed by staining with rhodamine phalloidin and immunostaining with anti-HA antibody to detect transfected cells. Data represent the percentage of cells without stress fibers per total number of transfected cells taken as 100%. Data represent mean values ± SE by ANOVA (Bonferroni Dunn). Scale bar, 20 µm.

Because loss of TSC2 upregulates mTOR activity, we examined whether TSC2-dependent stress fiber remodeling and migration is mediated by mTOR. mTOR forms two distinct complexes: the rapamycin-sensitive mTORC1 (mTOR-Raptor) and the rapamycin-insensitive mTORC2 (mTOR-Rictor), known to be involved in the regulation of the actin cytoskeleton [16], [17]. In agreement with our previous study showing that mTORC1 inhibitor rapamycin had little effect on migration of human LAM-derived cells [7], we found no significant differences in migration of *Tsc2* ^−/−^ MEFs treated for 4 hours with 20 nM rapamycin or diluent (0.91±0.01 fold vs. 1 fold for diluent-treated cells), suggesting that TSC2 regulates cell migration independently of mTORC1. We performed a co-microinjection of TSC2-null ELT3 cells with eitherkinase-dead mTOR (mTOR-KD), specific siRNA mTOR, siRNA Rictor, siRNA Raptor, or control siRNA, and GFP to identify microinjected cells, followed by rhodamine-phalloidin staining to assess the effects of mTOR, Rictor and Raptor on the actin cytoskeleton. We found that siRNA-induced down-regulation of mTOR or overexpression of mTOR-KD promoted stress fiber disassembly in TSC2-null ELT3 cells (Fig. 7 A–C). Importantly, siRNA-dependent knock-down of Rictor promoted stress fiber disassembly, while siRNA Raptor had no significant effect on stress fibers compared to GFP-injected cells (Fig. 7A, B).

**Figure 8:**
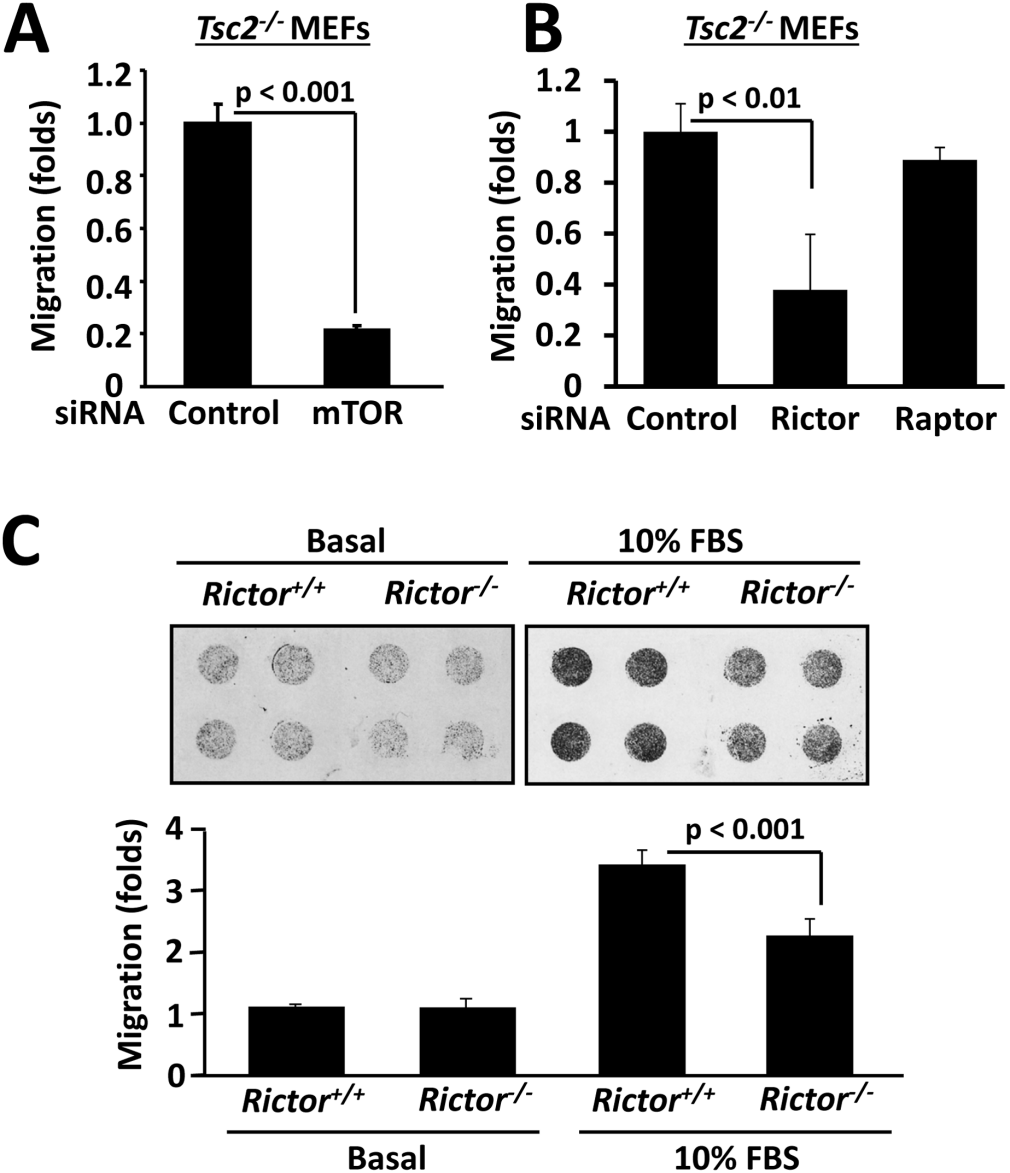
mTOR and Rictor mediate cell migration. A: siRNA-induced mTOR depletion inhibits *Tsc2^−/−^* MEF migration. *Tsc2^−/−^* MEFs were transfected with siRNA mTOR or control siRNA followed by migration assay. Migration of control siRNA-transfected cells was taken as 100%. B: Rictor is required for serum-induced MEF migration. *Tsc2^−/−^* MEFs were transfected with siRNA Rictor,siRNA Raptor, or control siRNA, and migration assays were subsequently performed. Migration of *Tsc2^−/−^* MEFs transfected with control siRNA was taken as 1 fold. C: Rictor is required for cell migration under nutrient-replete conditions but is not sufficient to modulate all migration. Migration assays were performed with *Rictor^+/+^* and *Rictor^−/−^* MEFs under basal (unstimulated) and serum-stimulated (10% FBS) conditions. Migration of *Rictor^+/+^* MEFs under basal conditions was taken as 100%.

Further, siRNA mTOR inhibited the migration of *Tsc2^−/−^* MEFs (Fig. 8A) demonstrating that mTOR is required for stress fiber assembly and increased cell migration due to TSC2 loss. siRNA Rictor, but not siRNA Raptor, attenuated migration of *Tsc2^−/−^* MEFs (Fig. 8B), showing that Rictor is required for TSC2-dependent MEF migration. Interestingly, the migrations of *Rictor^−/−^* and wild type *Rictor^+/+^* MEFs were comparable under basal unstimulated conditions (Fig. 8C, left panel) indicating that loss of Rictor in TSC2-expressing cells is not sufficient to modulate cell migration. However, in serum-replete conditions, migration of *Rictor^−/−^* MEFs was significantly decreased compared to wild type cells suggesting a Rictor requirement for cell migration under nutrient-replete conditions. Collectively, our data demonstrate that mTOR and Rictor, components of rapamycin-insensitive mTORC2, but not Raptor, a member rapamycin-sensitive mTORC1, are involved in the regulation of the actin cytoskeleton and cell migration, and mTORC2 is required for TSC2-related phenotypes as seen in TSC2^−/−^ MEFs.

## Discussion

In this study, we demonstrate differential roles of TSC1 and TSC2 in cell migration, invasiveness, and actin cytoskeleton. We show that TSC2 deficiency promotes cell migration and invasiveness while loss of TSC1 attenuates cell migration and reduces cellular invasive potential. We also report that TSC1 and TSC2 loss have differential effects on actin cytoskeleton organization and cell morphology and that TSC2-dependent alterations in the actin cytoskeleton and cell migration are modulated in an mTORC2-specific manner. Our findings show that TSC1 is required for cell migration but TSC2 suppresses cell migration, and suggest a potential link between TSC2 and mTORC2 in modulating the actin cytoskeleton and cell migration.

Alterations in the actin cytoskeleton and cell migration are seen during the progression of many human diseases, especially cancer. While at present LAM, a rare pulmonary disease, is not classified as a cancer, genetic and experimental data demonstrate that LAM cells display certain characteristics of cancerous cells including aberrant migration and invasiveness [7], extravasation into the blood and lymphatic circulatory systems [30], [31], and colonization of distant organs [8]. Studies on animal models show that the naturally occurring TSC2 mutations and TSC2 loss of heterozygosity (LOH) in the Eker rat leads to renal adenoma and carcinoma, some of which become malignant and metastasize to the lung, pancreas, and liver [32]. The hematogenous lung metastases of malignant uterine leiomyosarcoma are also identified in the Eker rat [33]. TSC2-null ELT3 smooth muscle cells derived from Eker rat uterine leiomyomas develop tumors in nude mice [34], and *Tsc2^−/−^* rat embryonic fibroblasts show capacity for anchorage-independent growth, one of the hallmarks of the invasive cell phenotype [35]. Furthermore, identical *TSC2* mutations were found in LAM cells in lungs and AML cells from renal tumors of TSC patients with LAM [36], and formation of secondary tumors with identical *TSC2* mutations has been reported in the lymph nodes of LAM patients [37]. Additionally, LAM nodule recurrence was reported after single-lung transplantation in patients without renal AML [8]. Disseminated neoplastic LAM cells with TSC2 loss are detected in the blood and chylous fluid of LAM patients [30]. Primary cultures of LAM-derived cells show increased motility and invasiveness, which is inhibited by TSC2 expression [7]. These evidences support the notion that TSC2 loss of function may manifest in inappropriate migratory and invasive cell characteristics. To date, however, there is no evidence linking TSC1 loss or mutational inactivation with LAM and/or TSC pro-metastatic phenotype.

Interestingly, *TSC*2 mutations occur more frequently than *TSC1* mutations in TSC and LAM, and mutational inactivation of TSC2 is predominantly associated with higher disease severity [38] suggesting different impacts of TSC1 or TSC2 loss on disease progression [2], [3]. Our study demonstrates that TSC1 and TSC2 loss have opposite effects on cell migration and invasiveness, two important components of the pro-metastatic cell phenotype. Using a combination of *Tsc1^−/−^, Tsc2^−/−^* and matched *Tsc1^+/+^, Tsc2^+/+^* MEF models and siRNA- and mammalian vector-based approaches, we demonstrate that TSC1 and TSC2 differentially regulate cell migration and invasiveness, and have different effects on actin cytoskeleton organization and cell morphology. Thus, *Tsc1^−/−^* MEFs have decreased migration and invasiveness, while migration and invasive potential of *Tsc2^−/−^* MEFs are markedly increased compared to littermate-matched controls. Furthermore, re-expression of TSC1 and TSC2 in *Tsc1^−/−^* and *Tsc2^−/−^* cells, respectively, rescues the wild-type phenotype, while siRNA-induced TSC1 and TSC2 knock-down respectively reduces and increases migration of wild-type MEFs and NIH 3T3 fibroblasts. Together with our data on different effects of TSC1 and TSC2 on cell shape and actin dynamics, these findings suggest that TSC2-dependent modulation of cell morphology, actin cytoskeleton, and migration might by modifying factors in disease severity attributed to TSC2 mutations in LAM and TSC.

TSC1 and TSC2 form a membrane-bound tumor suppressor complex, in which TSC1 functions as the regulatory component stabilizing TSC2 and facilitating the catalytic activity of TSC2 as a GAP for the small GTPase Rheb, a positive regulator of mTORC1, cell growth and proliferation [10], [11]. Upon growth-promoting stimuli, TSC2 dissociates from the membrane-bound complex and translocates to the cytosol [39]. TSC1 apparently remains at the membrane by binding to the ERM family of actin-binding proteins which is critical for RhoA activation by TSC1 and TSC1-dependent regulation of the actin cytoskeleton [13]. We previously demonstrated that TSC1 acts downstream of TSC2 in modulating RhoA activity, and loss of TSC2 results in TSC1-induced activation of RhoA and Rac1 inhibition [14]. We also reported that TSC2-RhoA signaling plays a role in cell migration and invasiveness, which may contribute to the pathobiology of LAM [7]. In our preclinical studies, therapeutic targeting of Rho GTPase in Tsc2-null cells, tumors and lung lesions induces cell apoptosis and prevents Tsc2-null tumor recurrence thus identifying simvastatin as a potential drug for diseases are associated with TSC2 deficiency [18], [40]–[42]. Specific mechanisms of TSC2-dependent cell migration, however, are poorly understood [7].

mTOR forms a catalytic core of two functionally distinct complexes, mTORC1 and mTORC2, which differ by their sensitivity to rapamycin. mTORC1 is acutely sensitive to rapamycin and controls protein translational regulation. mTORC2 is rapamycin insensitive in many cell types including TSC2-deficient and smooth muscle-like LAM cells and controls actin cytoskeleton [16], [17]. mTOR regulates the actin cytoskeleton through Rac1 [16], [17] as a part of the rapamycin-insensitive complex [43]. Our published data demonstrate that TSC2 loss induces mTORC2-specific up-regulation of RhoA that is mTORC1-independent and is required for TSC2-null lesion growth in mice [18]. In this study, our data show requirement of mTOR and mTORC2-specific Rictor, but not mTORC1-specific Raptor, for stress fiber formation and increased migration of Tsc2^−/−^ MEFs, strongly suggesting that increased cell migration due to TSC2 loss is mTORC2-dependent. Furthermore, effects of mTOR and Rictor on actin cytoskeleton disassembly suggest potential involvement of small GTPases RhoA and Rac1, downstream effectors of TSC2 and established controllers of actin remodeling. Together with published studies, our data suggest a new mTORC2-specific mechanism by which TSC2 loss or dysfunction may increase cellular pro-metastatic potential, but further studies are needed to elucidate whether cross-talk exists among mTORC2, RhoA and Rac1 in regulating actin rearrangements and cell migration due to TSC2 loss.

Collectively, this study demonstrates that TSC1 and TSC2 differentially regulate cell migration. While TSC1 is required for maintenance of actin filaments and cell migration, TSC2 induces actin filament disassembly and suppresses cell migration in mTORC2-specific manner. TSC2 loss leads to the development of a migratory and invasive cell phenotype that may contribute to the neoplastic nature of pulmonary LAM. Further studies of specific mechanisms by which TSC1 and TSC2 regulate cell migration and invasiveness may be beneficial for better understanding of LAM and TSC pathogenesis and development of novel therapeutic approaches.
