# Correction: Tumor Suppressors TSC1 and TSC2 Differentially Modulate Actin Cytoskeleton and Motility of Mouse Embryonic Fibroblasts

**DOI:** 10.1371/journal.pone.0118637

**Published:** 2015-03-05

**Authors:** The PLOS ONE Staff

The RO1 HL113178 grant is missing from the Funding section.

The complete, correct Funding section is: This work is supported by grants from the NIH/NHLBI RO1 HL090829 (V.P.K.), 2RO1 HL071106 (V.P.K.), RO1 HL114085 (V.P.K.),and RO1 HL113178 (E.A.G.), DOD IDEA TS030012 (V.P.K.), the LAM Foundation (V.P.K. and E.A.G.), and the American Thoracic Society (E.A.G.). The funders had no role in study design, data collection and analysis, decision to publish, or preparation of the manuscript.
